# Supplementary material for: Modeling early pathophysiological phenotypes of diabetic retinopathy in a human inner blood-retinal barrier-on-a-chip
Source: Nat Commun. 2024 Feb 14;15:1372. doi: 10.1038/s41467-024-45456-z (PMC10866954; doi:10.1038/s41467-024-45456-z)
Supplement: Supplementary file 1 — Supplementary Information [file 41467_2024_45456_MOESM1_ESM.pdf]

Supplementary information

Modeling diabetic retinopathy early pathophysiological phenotypes in a human inner blood-retinal barrier-on-a-chip

Maurissen et al.

Contents

Supplementary Fig. 1 | Characterization of iBRB MVN morphology.

Supplementary Fig. 2 | Donor heterogeneity on iBRB MVN formation.

Supplementary Fig. 3 | Characterization of iBRB formation.

Supplementary Fig. 4 | Characterization of iBRB maturity.

Supplementary Fig. 5 | Chronic diabetic treatment causes vascular regression.

Supplementary Fig. 6 | Chronic diabetic treatment causes reduced perivascular coverage.

Supplementary Fig. 7 | 3D analysis confirms diabetic phenotypic changes.

Supplementary Fig. 8 | Effects of diabetic cocktail components.

Supplementary Fig. 9 | Cell viability assays.

Supplementary Fig. 10 | Sequence of cell death events.

Supplementary Fig. 11 | Perfusability and permeability assays.

Supplementary Fig. 12 | RNA-sequencing analysis.

Supplementary Fig. 13 | Gene set enrichment analysis.

Supplementary Fig. 14 | Pathway analysis.

Supplementary Fig. 15 | Diabetic analyte measurements.

Supplementary Fig. 16 | Inhibition of pericyte-endothelial cell communication pathways cause microvascular alterations.

Supplementary Fig. 17 | Long-term treatment effects.

Supplementary Fig. 18 | Treatments analyte measurements.

Supplementary Table 1 | Information about cells used in this study.

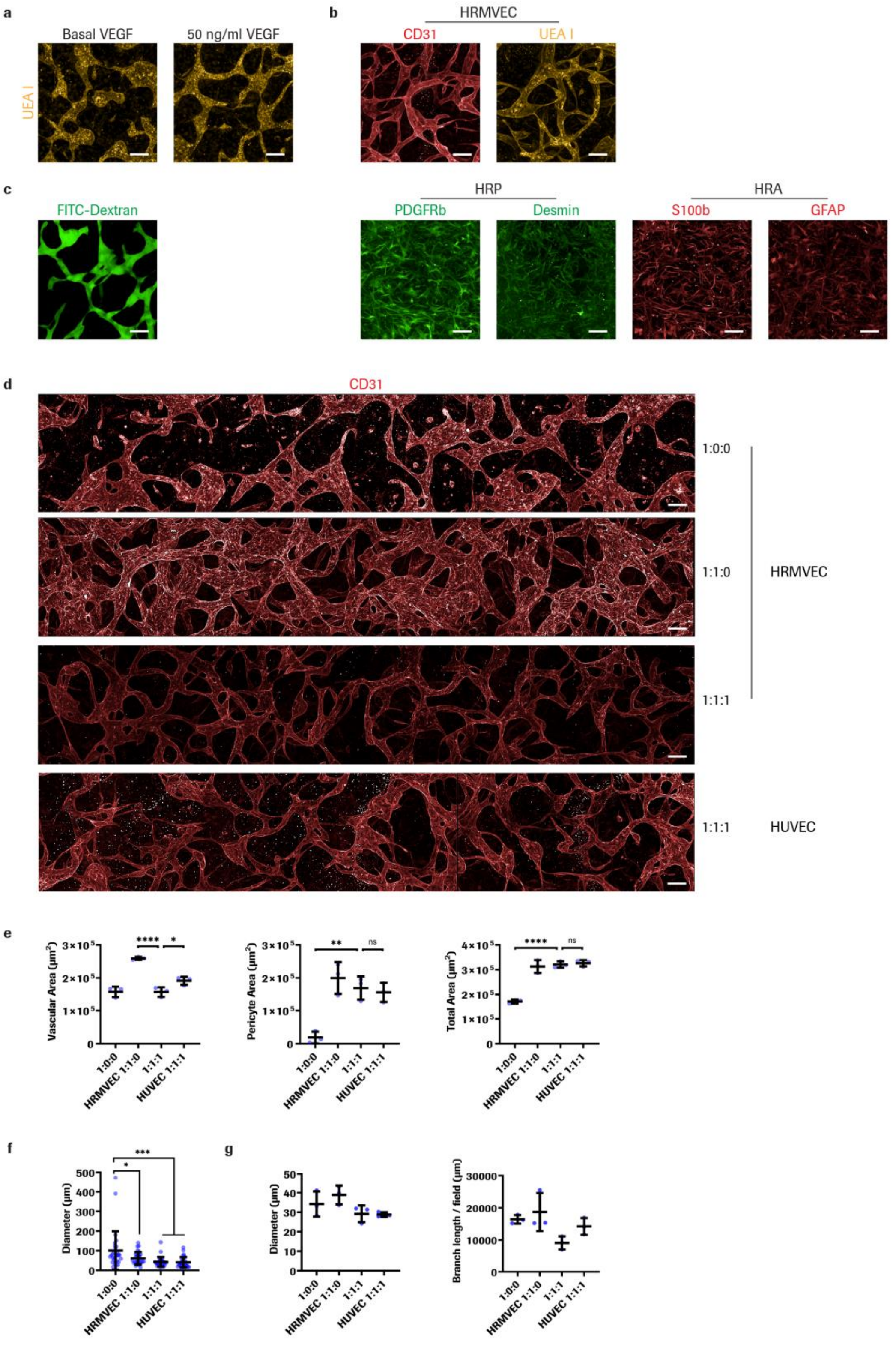
a
Basal VEGF
50 ng/ml VEGF
UEA I
b
HRMVEC
CD31
UEA I
c
FITC-Dextran
HRP
PDGFRb
Desmin
HRA
S100b
GFAP
d
CD31
1:0:0
1:1:0
1:1:1
HRMVEC
1:1:1
HUVEC
e
Vascular Area (µm²)
Pericyte Area (µm²)
Total Area (µm²)
****
*
**
ns
1:0:0
HRMVEC 1:1:0
1:1:1
HUVEC 1:1:1
f
Diameter (µm)
***
g
Diameter (µm)
Branch length / field (µm)

Supplementary Fig. 1 | Characterization of iBRB MVN morphology. a, Representative images of endothelial networks (UEA I) cultured in medium with basal (5 ng ml^{-1}) VEGF (left) or in medium supplemented with 50 ng ml^{-1} VEGF (right). **b**, Representative images of endothelial (HRMVEC) markers CD31 and UEA I (top), pericyte (HRP) markers PDGFRβ and Desmin (bottom left) and astrocyte (HRA) markers S100b and GFAP (bottom right). Stainings were repeated in n = 3 independent experiments with similar results. **c**, Endothelial networks perfused with FITC-dextran (100 µg ml^{-1}, 10 kDa). **d**, Global images of different HRMVEC:HRP:HRA cell ratios: monoculture (1:0:0), co-culture with HRMVECs and HRPs (1:1:0) and tri-culture with HRMVECs, HRPs and HRAs (1:1:1). Tri-culture with HUVECs, HRPs and HRAs (1:1:1) is shown as comparison. **e**, Quantification of mean vascular, pericyte and total areas per channel. n = 3 HRMVEC 1:0:0, n = 3 1:1:0, n = 3 1:1:1 and n = 3 HUVEC 1:1:1 whole channels analyzed from n = 3 independent experiments. **f**, Distribution of vessel diameters for one whole channel per condition quantified manually. n = 6 HRMVEC 1:0:0, n = 6 1:1:0, n = 6 1:1:1 and n = 6 HUVEC 1:1:1 network ROI analyzed. **g**, Quantification of mean vessel diameter and branch length per channel. n = 3 HRMVEC 1:0:0, n = 3 1:1:0, n = 3 1:1:1 and n = 3 HUVEC 1:1:1 whole channels analyzed from n = 3 independent experiments. Data are mean ± s.d. *P<0.05; **P<0.01; ***P<0.001; ****P<0.0001; one-way ANOVA. Source data are provided as a Source Data file. All images show maximum intensity projections of 290 µm Z-stacks. Scale bars, 100 µm (**a**-**d**).

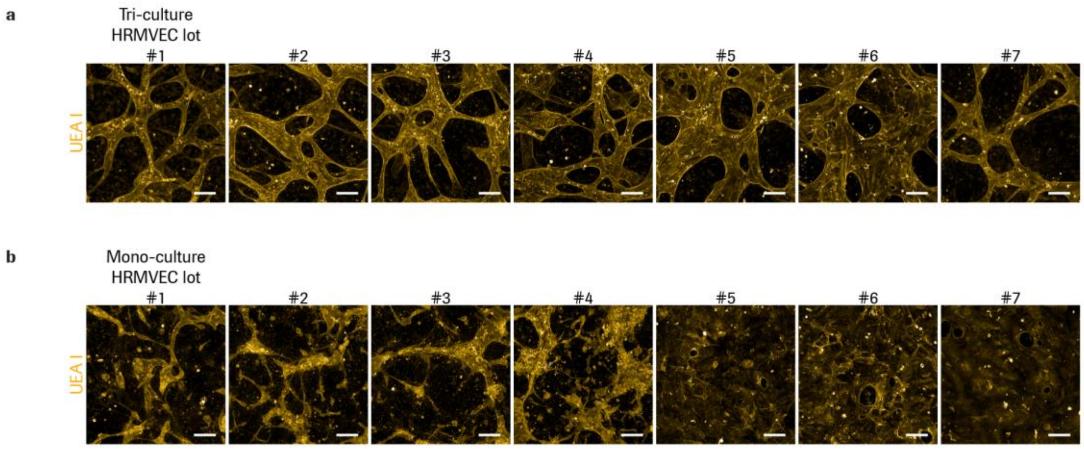

Supplementary Fig. 2 | Donor heterogeneity on iBRB MVN formation. a, Representative image of comparison between seven different lots of HRMVECs (UEA I) co-cultured with HRPs and HRAs. **b**, Comparison between different lots of HRMVECs (UEA I) in mono-culture. HRMVEC lots #5, #6 and #7 formed a monolayer. All images show maximum intensity projections of 290 µm Z-stacks. Scale bars, 100 µm (**a**, **b**).

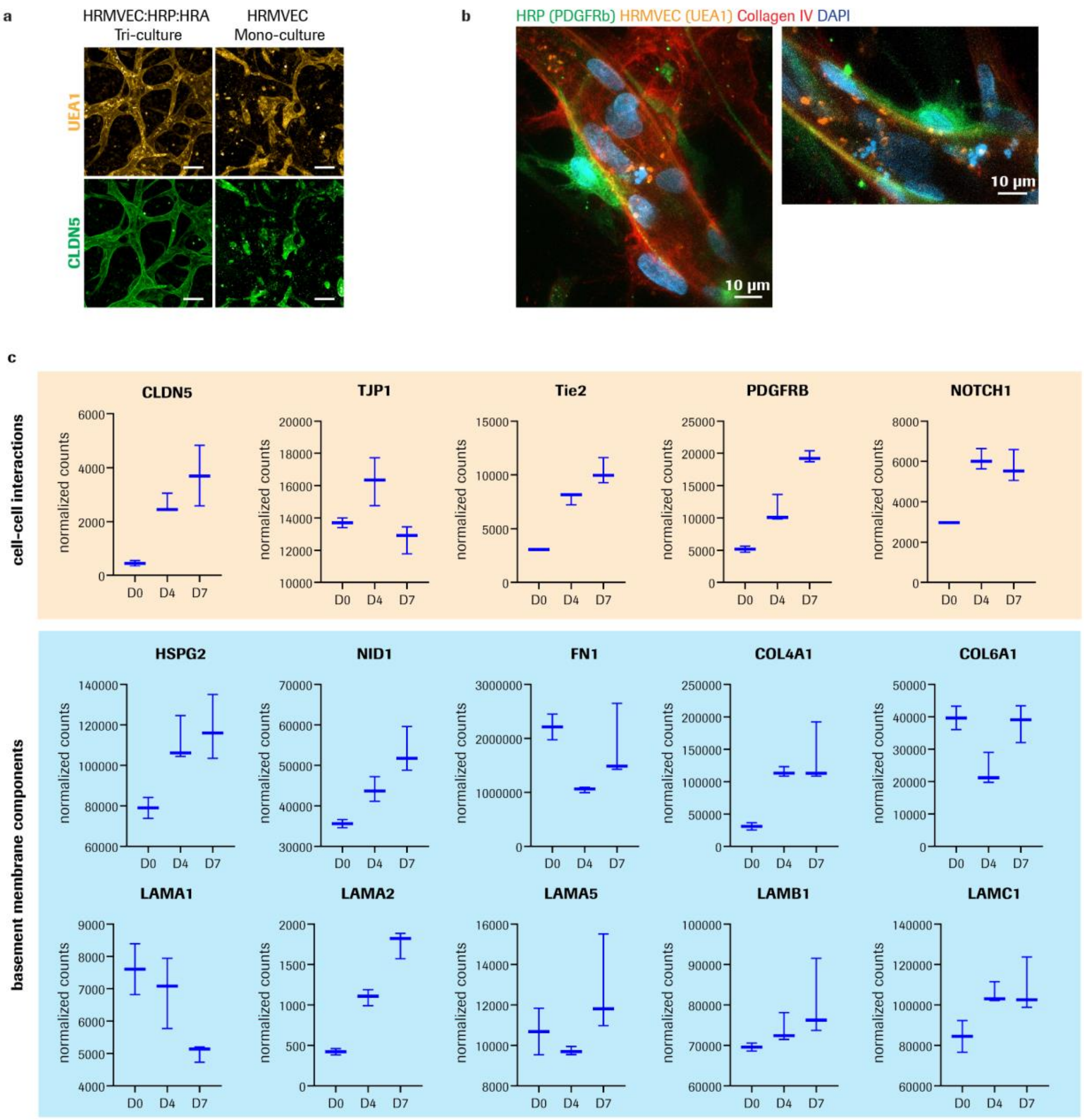

Supplementary Fig. 3 | Characterization of iBRB MVN formation. a, Representative image comparison between 3D mono- and tri-cultures, and tight junction (CLDN5) integrity. Images show maximum intensity projections of 290 μm Z-stacks. Scale bars, 100 μm. **b**, Confocal images at 63x magnification showing pericytes (PDGFRβ green) lining the vessels (UEA I orange). **c**, Normalized counts of genes involved in cell-cell interactions and basement membrane

composition during the maturation phase of the iBRB-on-a-chip (D0 to D7). Data are from n = 3 independent experiments.

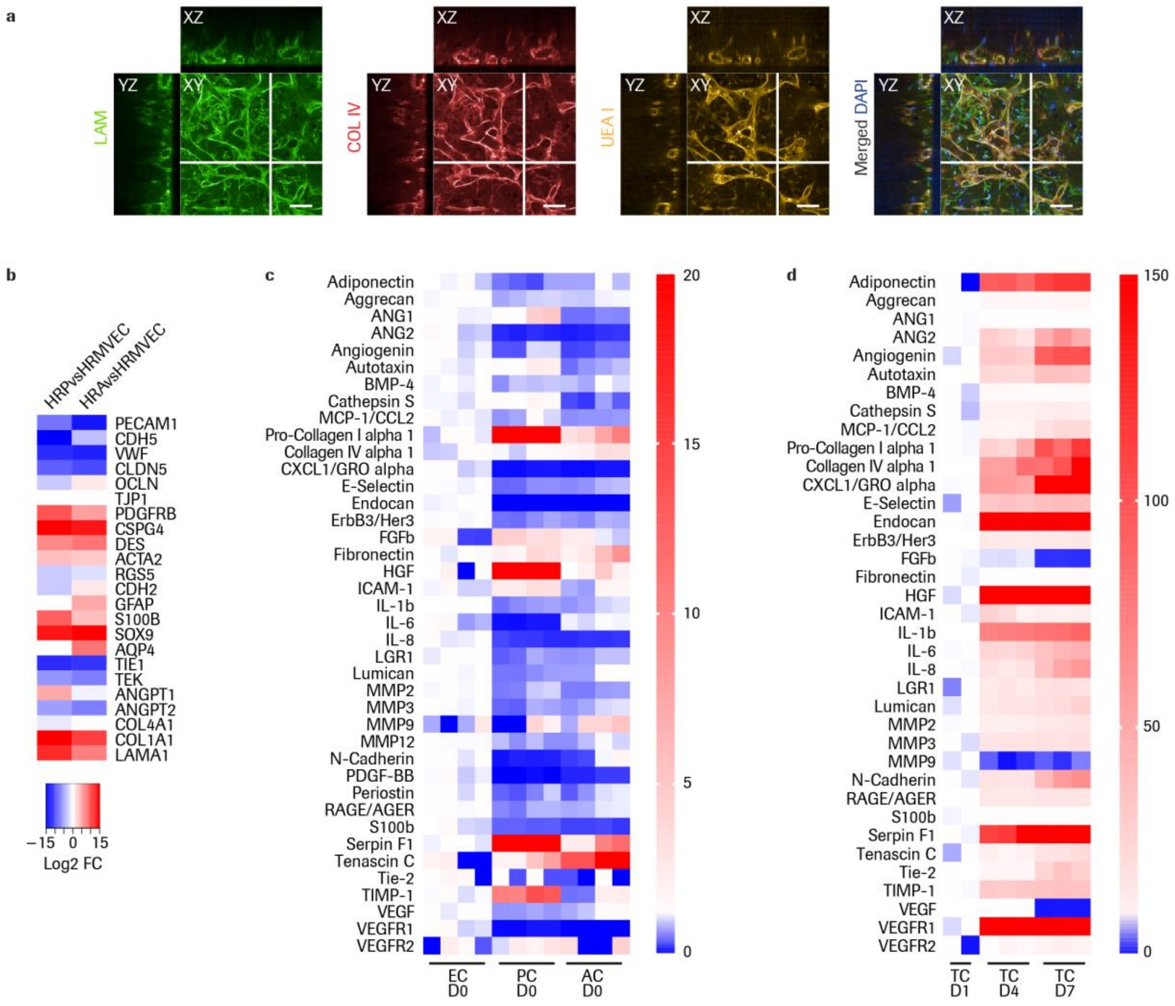

Supplementary Fig. 4 | Characterization of iBRB maturity. a, Representative cross-section images of basement membrane proteins (LAM and COL IV). Images show orthogonal projections of 290 µm Z-stacks. Stainings were repeated in n = 3 independent experiments with similar results. **b**, Heat map of differential gene expression between HRPs and HRMVECs, and between HRAs and HRMVECs on D0. Results are from a characterization gene panel without cutoff. Data are RNA-Seq aggregated Log2 FC from n = 3 independent experiments. Genes with a high Log2 FC (red) are upregulated in HRPs or HRAs while genes with a low Log2 FC (blue) are upregulated in HRMVECs. **c**, Heat map of analyte measurements on HRMVEC (EC), HRP

(PC) and HRA (AC) supernatants on D0, showing analyte concentrations normalized to the mean EC value on D0. **d**, Heat map of analyte measurements on tri-culture (TC) supernatants on D1, D4 and D7, normalized to the mean TC value on D1. The scale indicates fold change to control. Supernatants were obtained from n = 4 independent experiments. Source data are provided as a Source Data file. Scale bars, 100 μm (**a**).

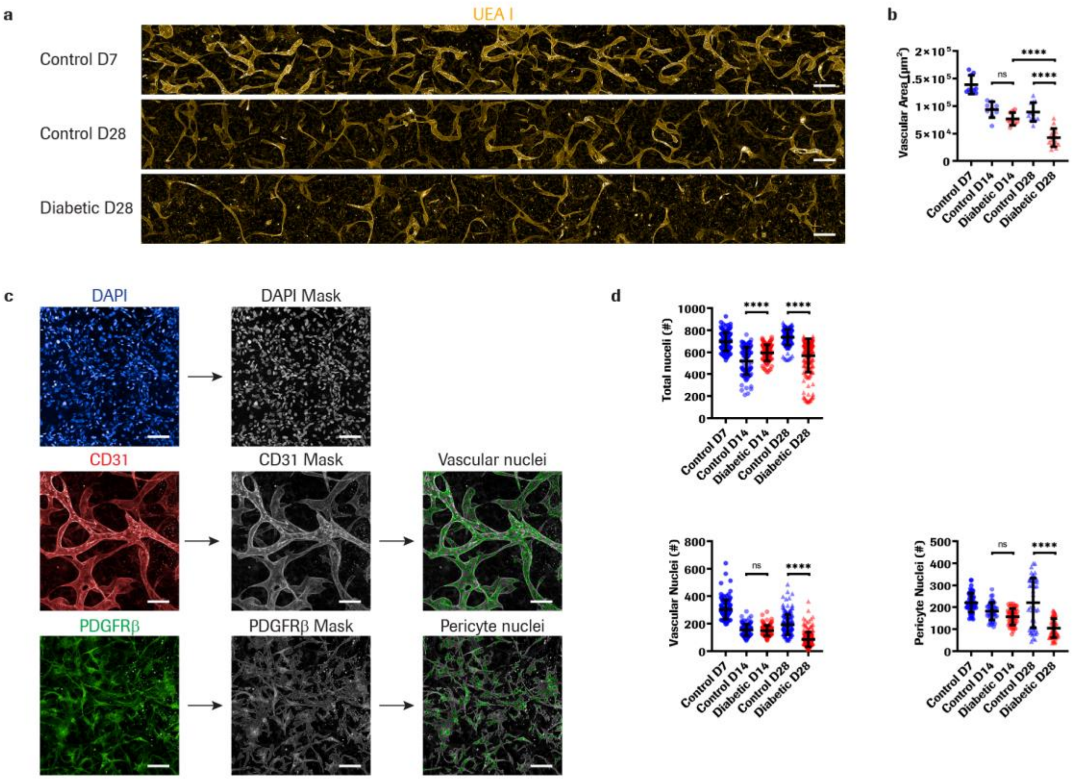

Supplementary Fig. 5 | Chronic diabetic treatment causes vascular regression. a, Representative channel images of iBRB MVNs (UEA I) cultured in normal medium on D7 (top), D28 (middle) or in diabetic medium on D28 (bottom). Vascular regression is visible in the diabetic condition on D28. **b**, Quantification of mean vascular area per channel. n = 9 control D7, n = 9 D14, n = 12 D28, n = 9 diabetic D14 and n = 15 D28 whole channels analyzed from n = 3 independent experiments. **c**, Image analysis pipeline of maximum intensity projections obtained from whole-channel image acquisitions with fluorescence confocal microscopy. Nuclei (DAPI) were segmented, and ECs (CD31) and pericytes (PDGFRβ) were thresholded to obtain masks and quantify respective areas. Nuclei included in each mask were also quantified. **d**, Quantification of the number of total (top), vascular (bottom left) and pericyte (bottom right) nuclei for control

conditions on D7, D14 and D28 and diabetic conditions on D14 and D28. n = 113 control D7, n = 117 D14, n = 165 D28, n = 117 diabetic D14 and n = 203 D28 ROI networks analyzed for total and vascular nuclei, and n = 49 control D7, n = 42 D14, n = 48 D28, n = 48 diabetic D14 and n = 61 D28 ROI networks analyzed for pericyte nuclei from n = 3 independent experiments. Data are mean ± s.d. ****$P<0.0001$; one-way ANOVA. Source data are provided as a Source Data file. Images show maximum intensity projections of 395 μm Z-stacks. Scale bars, 100 μm (**a**, **c**).

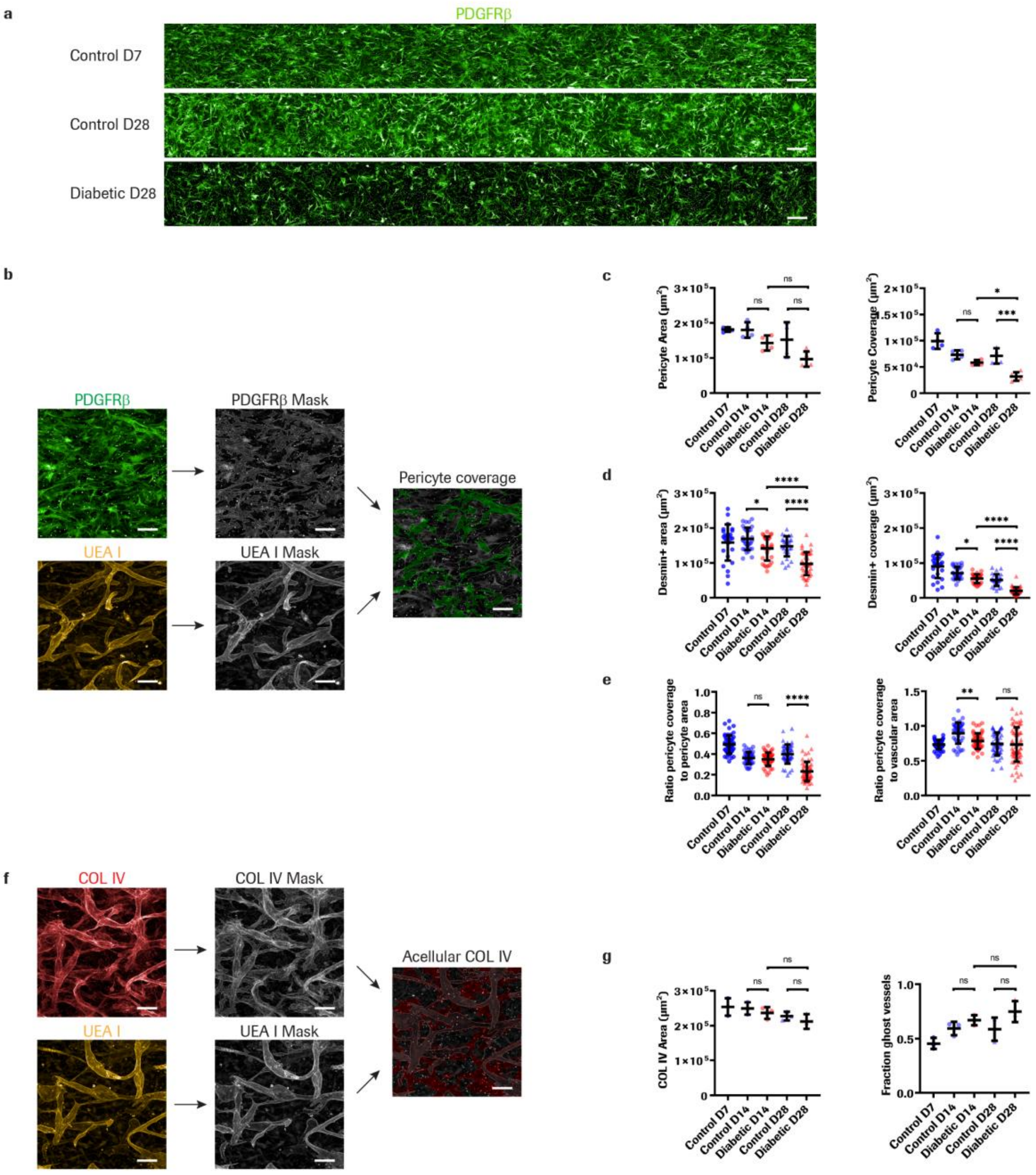

Supplementary Fig. 6 | Chronic diabetic treatment causes reduced perivascular coverage. a, Representative channel images of pericytes (PDGFRβ), with pericyte loss visible in the diabetic condition on D28. **b**, Image analysis for quantifying pericyte coverage. Maximum intensity projections of pericytes (PDGFRβ) and ECs (UEA I) were thresholded to obtain masks, and the

pericyte coverage was calculated from the overlapping areas. **c**, Quantification of mean pericyte area and coverage per channel. n = 4 control D7, n = 4 D14, n = 4 D28, n = 4 diabetic D14 and n = 5 D28 whole channels analyzed from n = 3 independent experiments. **d**, Quantification of pericyte area (left) and coverage (right) based on the pericyte-specific marker Desmin, for control conditions on D7, D14 and D28 and diabetic conditions on D14 and D28. The results are consistent with pericyte quantifications using the PDGFRβ marker. n = 26 control D7, n = 30 D14, n = 27 D28, n = 29 diabetic D14 and n = 40 D28 ROI networks analyzed from n = 2 independent experiments. **e**, Quantification of pericyte coverage to pericyte area ratio (left) and pericyte coverage to vascular area ratio (right). n = 49 control D7, n = 42 D14, n = 48 D28, n = 48 diabetic D14 and n = 61 D28 ROI networks analyzed from n = 3 independent experiments. **f**, Image analysis of ghost vessels. Maximum intensity projections of basement membrane (COL IV) and ECs (UEA I) were thresholded and ghost vessels were obtained by calculating the difference between COL IV and UEA I mask areas. **g**, Quantification of mean COL IV area and avascular area per channel. n = 3 control D7, n = 3 D14, n = 3 D28, n = 3 diabetic D14 and n = 3 D28 whole channels analyzed from n = 3 independent experiments. Data are mean ± s.d. *P<0.05; **P<0.01; ****P<0.0001; one-way ANOVA. Source data are provided as a Source Data file. Images show maximum intensity projections of 395 μm Z-stacks. Scale bars, 100 μm (**a**, **b**, **f**).

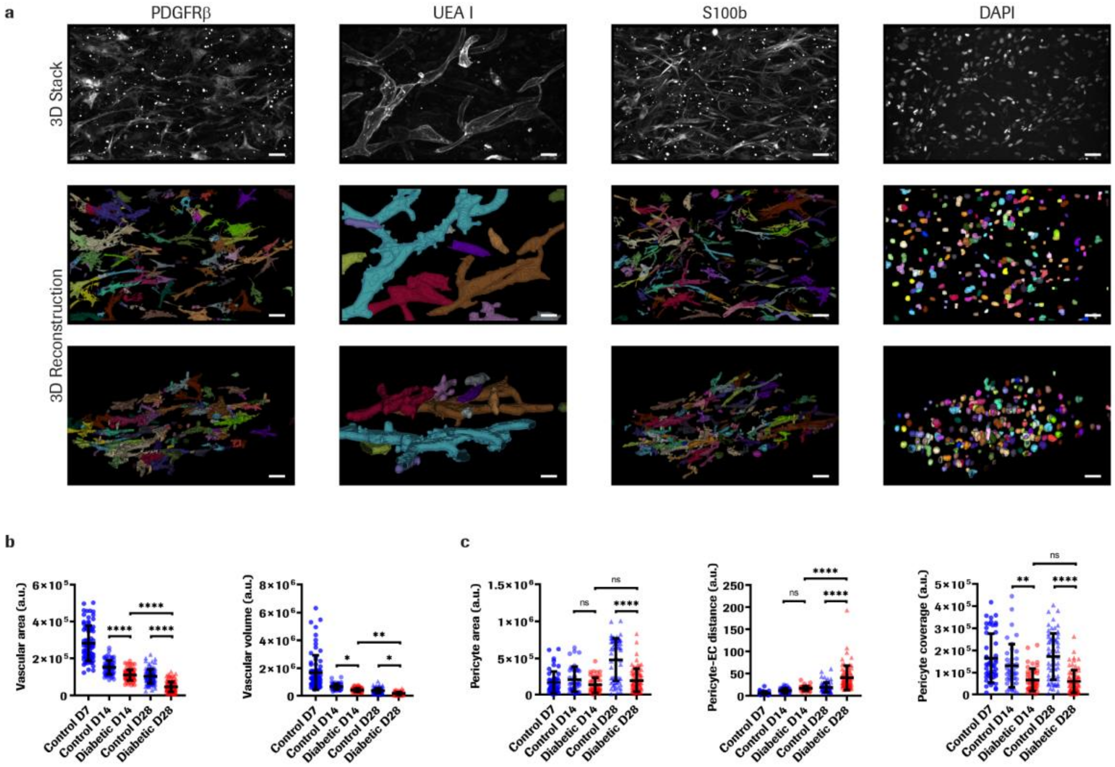

Supplementary Fig. 7 | 3D analysis confirms diabetic phenotypic changes. a, 3D image analysis through reconstruction and segmentation of pericyte- (PDGFRβ), EC- (UEA I), astrocyte- (S100b) and nuclei-specific (DAPI) stainings. Fluorescent image stacks (top), and different views of image reconstructions (middle and bottom) are visualized. Images show 3D views of 395 μm Z-stacks. **b**, 3D quantification of vascular area (left) and volume (right) for control conditions on D7, D14 and D28 and diabetic conditions on D14 and D28. n = 75 control D7, n = 72 D14, n = 105 D28, n = 77 diabetic D14 and n = 130 D28 ROI networks analyzed from n = 3 independent experiments. **c**, 3D quantification of pericyte are (left), and pericyte-EC distance (middle) and area (right). Direct pericyte-EC interactions are reduced with diabetic treatment. n = 49 control D7, n = 42 D14, n = 63 D28, n = 48 diabetic D14 and n = 76 D28 ROI networks analyzed from n = 3 independent experiments. Data are mean ± s.d. *P<0.05; **P<0.01;

****$P<0.0001$; one-way ANOVA. Source data are provided as a Source Data file. Images show maximum intensity projections of 395 μm Z-stacks. Scale bars, 100 μm (**a**, **d**).

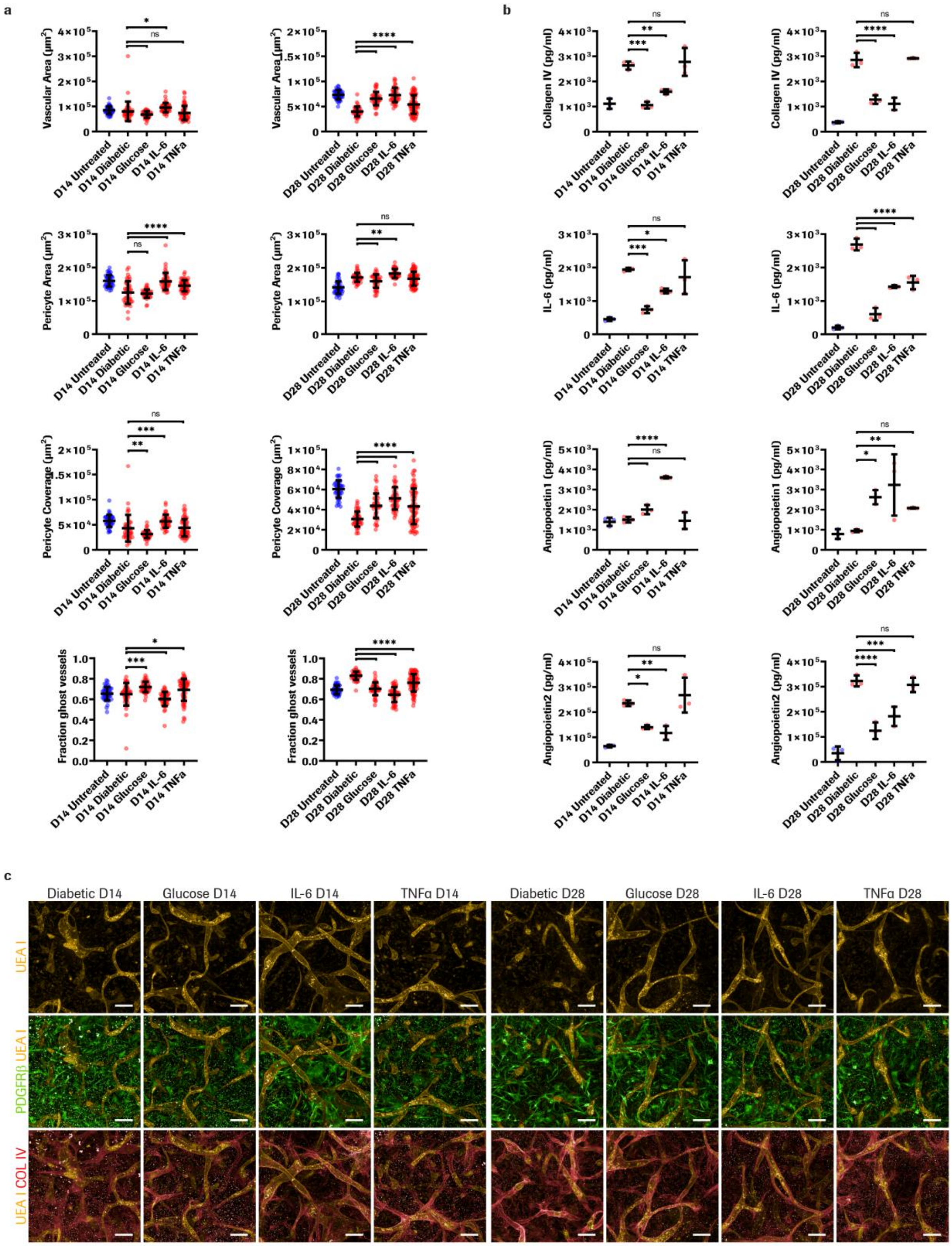

a
Vascular Area (µm²)
Pericyte Area (µm²)
Pericyte Coverage (µm²)
Fraction ghost vessels
D14 Untreated
D14 Diabetic
D14 Glucose
D14 IL-6
D14 TNFa
D28 Untreated
D28 Diabetic
D28 Glucose
D28 IL-6
D28 TNFa
b
Collagen IV (pg/ml)
IL-6 (pg/ml)
Angiopoietin1 (pg/ml)
Angiopoietin2 (pg/ml)
c
Diabetic D14
Glucose D14
IL-6 D14
TNFα D14
Diabetic D28
Glucose D28
IL-6 D28
TNFα D28
UEA I
PDGFRβ UEA I
UEA I COL IV

Supplementary Fig. 8 | Effects of diabetic cocktail components. a, Quantification of vascular area, pericyte area and coverage, and fraction ghost vessels for untreated, diabetic, glucose, IL-6 and TNF-α conditions on D14 and D28. n = 74 untreated D7, n = 45 D14, n = 44 D28, n = 44 diabetic D14, n = 43 D28, n = 44 glucose D14, n = 44 D28, n = 45 IL-6 D14, n = 44 D28, n = 59 TNF-α D14 and n = 75 D28 treated ROI networks analyzed from n = 3 replicate channels. **b**, Analyte measurements showing supernatant concentrations of human collagen IV alpha I, IL-6, Angiopoietin-1 and Angiopoietin-2 obtained from n = 3 replicate channels. Data are mean ± s.d. *P<0.05; **P<0.01; ***P<0.001; ****P<0.0001; one-way ANOVA. Source data are provided as a Source Data file. **c**, Representative images of endothelial networks (UEA I) and overlay images with pericytes (PDGFRβ) or basement membranes (COL IV) on D14 and D28, following 7 (D14) or 21 (D28) days of treatment respectively. Treatments with D-Glucose, IL-6 or TNF-α were tested individually, and in combination to produce the diabetic treatment (Diabetic). All images show maximum intensity projections of 245 μm Z-stacks. Scale bars, 100 μm (**c**).

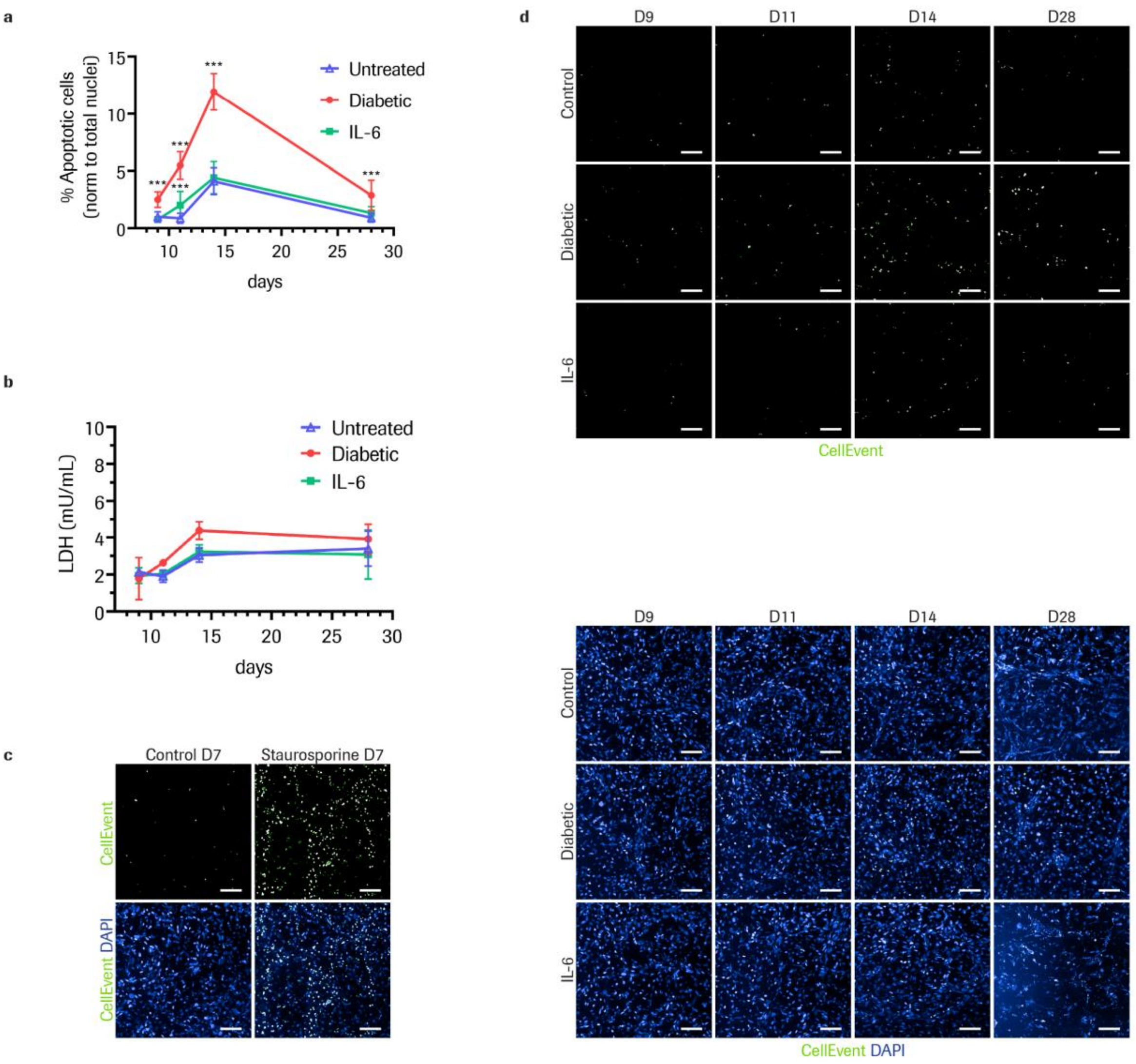

Supplementary Fig. 9 | Cell viability assays. a, Quantification of the percentage of apoptotic cells (CellEvent) normalized to total nuclei (DAPI) per field for untreated, diabetic and IL-6-treated conditions over time. n = 45 untreated D9, D11, D14 and D28, n = 45 diabetic D9 and D14, n = 44 diabetic D11 and D28, n = 45 IL-6 D9, D14 and D28, and n = 44 IL-6 D11 treated ROI networks analyzed from n = 3 replicate channels. **b**, Quantification of LDH release for untreated, diabetic and IL-6 conditions over time obtained from n = 3 replicate channels for each condition. Data are mean ± s.d. *P<0.05; **P<0.01; ***P<0.001; ****P<0.0001; one-way

ANOVA. Source data are provided as a Source Data file. **c**, Representative images of untreated control and Staurosporine-treated cells on D7. CellEvent marks apoptotic nuclei and DAPI all nuclei. Staurosporine is a positive control inducing apoptosis. **d**, Representative images of untreated control, diabetic cocktail, IL-6 alone, and Staurosporine control on D9, D11, D14 and D28. All images show maximum intensity projections of 245 μm Z-stacks. Scale bars, 100 μm (**c**, **d**).

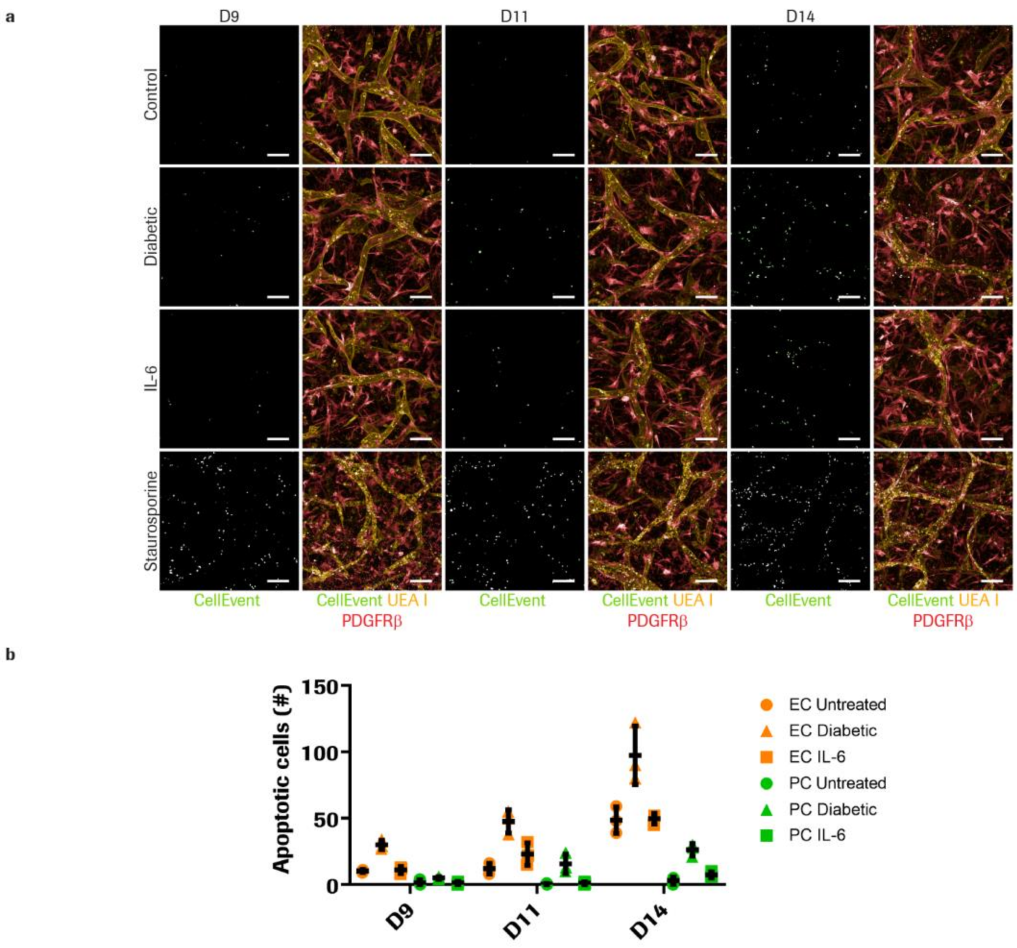

Supplementary Fig. 10 | Sequence of cell death events. a, Representative images of apoptotic nuclei (CellEvent), endothelial network (UEA I) and pericyte (PDGFRβ) stainings on D9, D11 and D14 for untreated control, diabetic, IL-6 and Staurosporine-treated conditions (positive control). **b**, Number of apoptotic cells by cell type; endothelial cells (EC) in orange and pericytes (PC) in green on D9, D11 and D14 for untreated control, diabetic and IL-6 conditions. One representative ROI per n = 3 replicate channels was quantified for each condition on D9, D11 and D14. Source data are provided as a Source Data file. All images show maximum intensity projections of 245 µm Z-stacks. Scale bars, 100 µm (**a**).

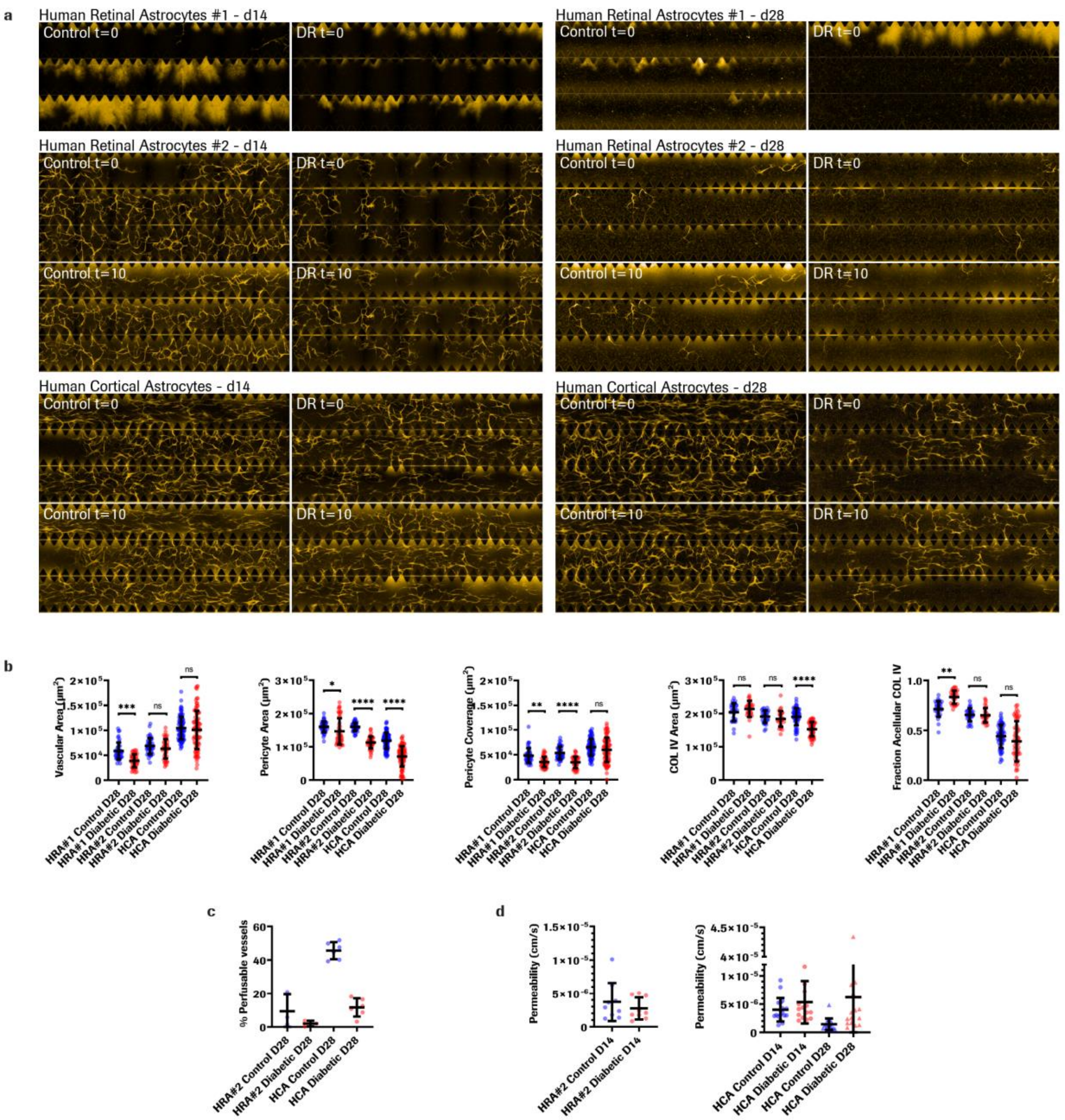

Supplementary Fig. 11 | Perfusability and permeability assays. a, Images of untreated control and diabetic iBRB MVNs perfused with TRITC-labelled 70kDa dextran at t = 0 and t = 10 min. Human retinal astrocytes (HRA) and human cortical astrocytes (HCA) from different donors were compared on D14 and D28. Triplicate channels are shown for each condition. Images show maximum intensity projections of 190 μm Z-stacks. Channel height visible is 1.3 mm. **b**,

Quantifications of vascular area, pericyte area and coverage, and COL IV area and avascular area. n = 45 HRA#1 untreated, n = 45 diabetic D28, n = 45 HRA#2 untreated, n = 45 diabetic D28, n = 91 HCA untreated, n = 90 diabetic D28 treated ROI networks analyzed from n = 3 replicate channels. n = 91 HCA diabetic D28 treated networks for pericyte area. n = 30 HRA#1 untreated, n = 30 diabetic D28, n = 30 HRA#2 untreated, n = 30 diabetic D28, n = 60 HCA untreated, n = 58 diabetic D28 treated ROI networks for COL IV area and avascular area. Data are mean ± s.d. *$P<0.05$; **$P<0.01$; ***$P<0.001$; ****$P<0.0001$; one-way ANOVA. **c**, Perfusability corresponding to the fraction of TRITC+ perfused vessels to UEA I+ total vessels on D28 for n = 3 HRA#2 and n = 6 HCA replicate channels per condition. **d**, Permeability values of perfusable untreated and diabetic MVNs containing HRA#2 or HCA in tri-cultures. n = 9 HRA#2 (3 channels), and n = 18 HCA (6 channels) network ROI analyzed per condition. Source data are provided as a Source Data file.

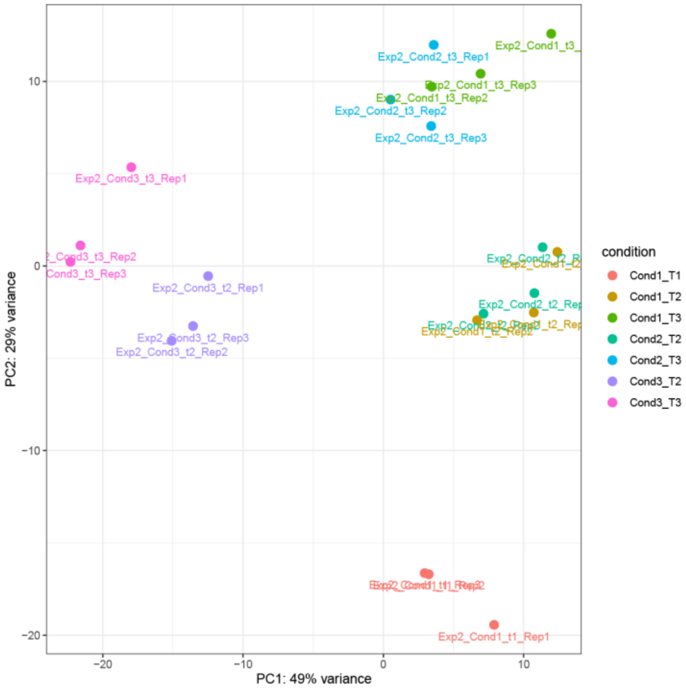

Supplementary Fig. 12 | RNA-sequencing analysis. Principal component analysis of RNA-sequencing replicate samples from n = 3 independent experiments. Cond1, untreated control; Cond2, osmotic control; Cond3, diabetic condition; T1, D7; T2, D14; T3, D28; Rep1-3, replicate 1-3.

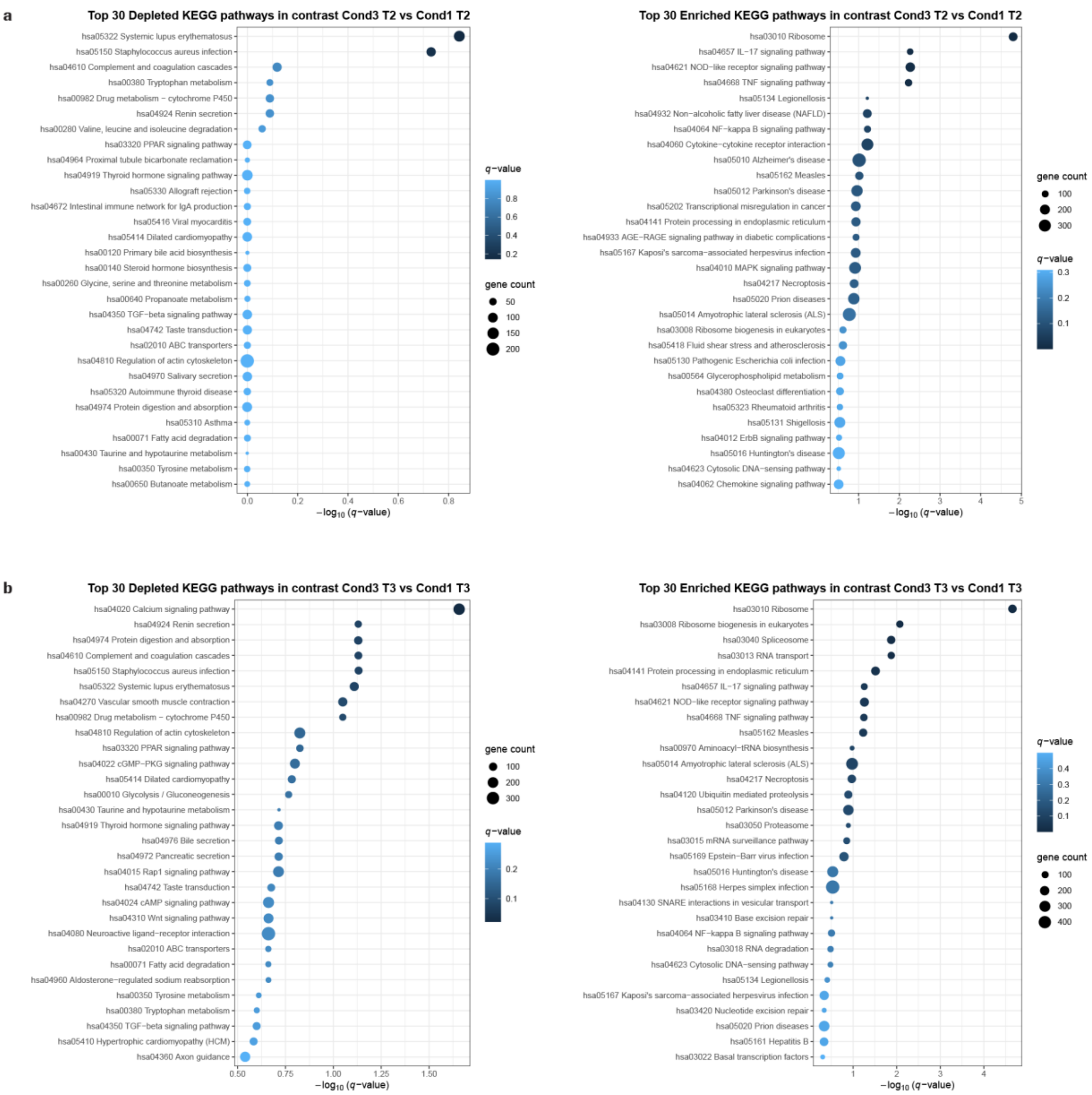

Supplementary Fig. 13 | Gene set enrichment analysis. a, Top 30 differentially regulated KEGG pathways between the diabetic condition and untreated control on D14. **b**, Top 30 KEGG pathways on D28.

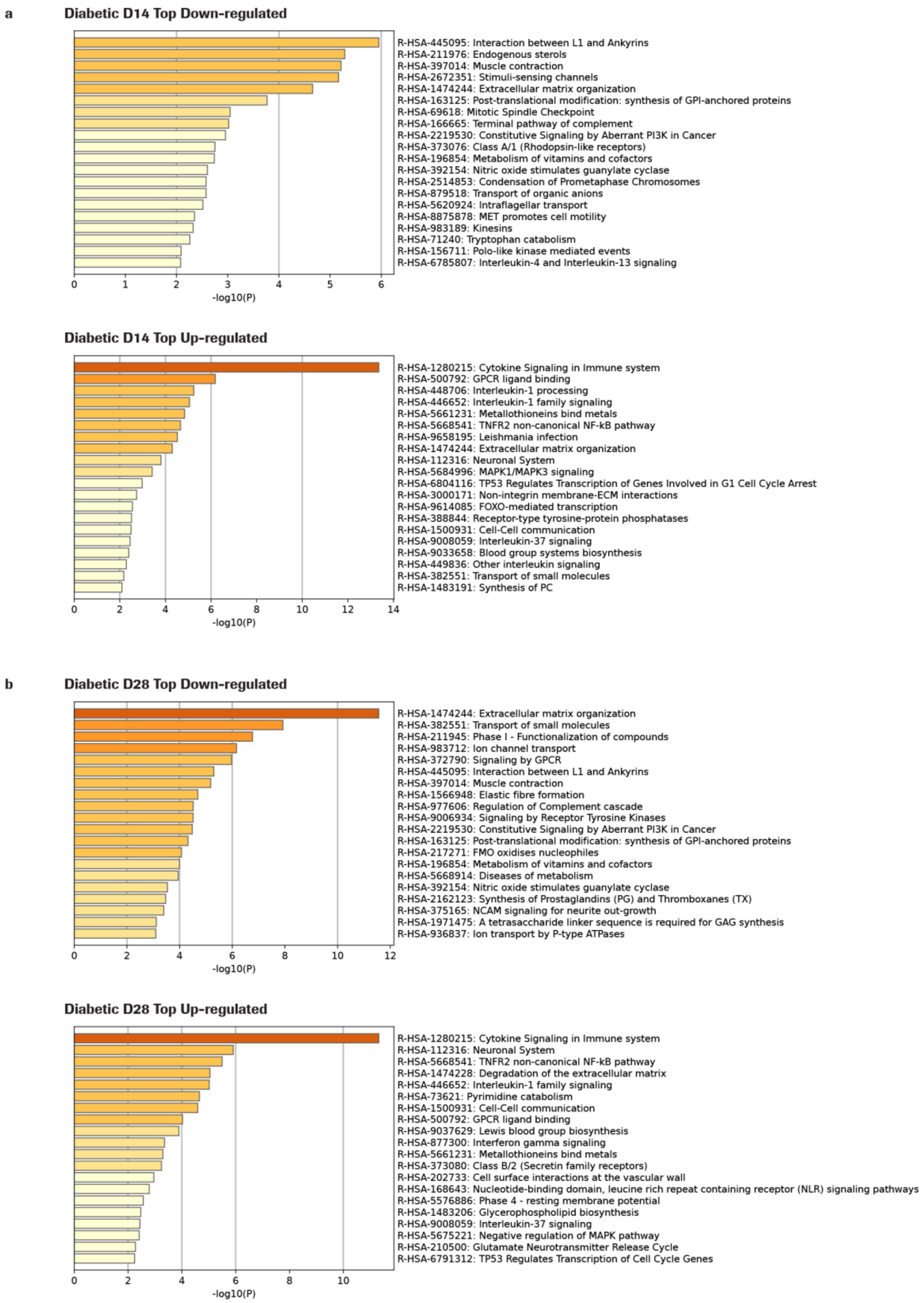

a
Diabetic D14 Top Down-regulated
R-HSA-445095: Interaction between L1 and Ankyrins
R-HSA-211976: Endogenous sterols
R-HSA-397014: Muscle contraction
R-HSA-2672351: Stimuli-sensing channels
R-HSA-1474244: Extracellular matrix organization
R-HSA-163125: Post-translational modification: synthesis of GPI-anchored proteins
R-HSA-69618: Mitotic Spindle Checkpoint
R-HSA-166665: Terminal pathway of complement
R-HSA-2219530: Constitutive Signaling by Aberrant PI3K in Cancer
R-HSA-373076: Class A/1 (Rhodopsin-like receptors)
R-HSA-196854: Metabolism of vitamins and cofactors
R-HSA-392154: Nitric oxide stimulates guanylate cyclase
R-HSA-2514853: Condensation of Prometaphase Chromosomes
R-HSA-879518: Transport of organic anions
R-HSA-5620924: Intraflagellar transport
R-HSA-8875878: MET promotes cell motility
R-HSA-983189: Kinesins
R-HSA-71240: Tryptophan catabolism
R-HSA-156711: Polo-like kinase mediated events
R-HSA-6785807: Interleukin-4 and Interleukin-13 signaling
0 1 2 3 4 5 6
-log10(P)
Diabetic D14 Top Up-regulated
R-HSA-1280215: Cytokine Signaling in Immune system
R-HSA-500792: GPCR ligand binding
R-HSA-448706: Interleukin-1 processing
R-HSA-446652: Interleukin-1 family signaling
R-HSA-5661231: Metallothioneins bind metals
R-HSA-5668541: TNFR2 non-canonical NF-kB pathway
R-HSA-9658195: Leishmania infection
R-HSA-1474244: Extracellular matrix organization
R-HSA-112316: Neuronal System
R-HSA-5684996: MAPK1/MAPK3 signaling
R-HSA-6804116: TP53 Regulates Transcription of Genes Involved in G1 Cell Cycle Arrest
R-HSA-3000171: Non-integrin membrane-ECM interactions
R-HSA-9614085: FOXO-mediated transcription
R-HSA-388844: Receptor-type tyrosine-protein phosphatases
R-HSA-1500931: Cell-Cell communication
R-HSA-9008059: Interleukin-37 signaling
R-HSA-9033658: Blood group systems biosynthesis
R-HSA-449836: Other interleukin signaling
R-HSA-382551: Transport of small molecules
R-HSA-1483191: Synthesis of PC
0 2 4 6 8 10 12 14
-log10(P)
b
Diabetic D28 Top Down-regulated
R-HSA-1474244: Extracellular matrix organization
R-HSA-382551: Transport of small molecules
R-HSA-211945: Phase I - Functionalization of compounds
R-HSA-983712: Ion channel transport
R-HSA-372790: Signaling by GPCR
R-HSA-445095: Interaction between L1 and Ankyrins
R-HSA-397014: Muscle contraction
R-HSA-1566948: Elastic fibre formation
R-HSA-977606: Regulation of Complement cascade
R-HSA-9006934: Signaling by Receptor Tyrosine Kinases
R-HSA-2219530: Constitutive Signaling by Aberrant PI3K in Cancer
R-HSA-163125: Post-translational modification: synthesis of GPI-anchored proteins
R-HSA-217271: FMO oxidises nucleophiles
R-HSA-196854: Metabolism of vitamins and cofactors
R-HSA-5668914: Diseases of metabolism
R-HSA-392154: Nitric oxide stimulates guanylate cyclase
R-HSA-2162123: Synthesis of Prostaglandins (PG) and Thromboxanes (TX)
R-HSA-375165: NCAM signaling for neurite out-growth
R-HSA-1971475: A tetrasaccharide linker sequence is required for GAG synthesis
R-HSA-936837: Ion transport by P-type ATPases
0 2 4 6 8 10 12
-log10(P)
Diabetic D28 Top Up-regulated
R-HSA-1280215: Cytokine Signaling in Immune system
R-HSA-112316: Neuronal System
R-HSA-5668541: TNFR2 non-canonical NF-kB pathway
R-HSA-1474228: Degradation of the extracellular matrix
R-HSA-446652: Interleukin-1 family signaling
R-HSA-73621: Pyrimidine catabolism
R-HSA-1500931: Cell-Cell communication
R-HSA-500792: GPCR ligand binding
R-HSA-9037629: Lewis blood group biosynthesis
R-HSA-877300: Interferon gamma signaling
R-HSA-5661231: Metallothioneins bind metals
R-HSA-373080: Class B/2 (Secretin family receptors)
R-HSA-202733: Cell surface interactions at the vascular wall
R-HSA-168643: Nucleotide-binding domain, leucine rich repeat containing receptor (NLR) signaling pathways
R-HSA-5576886: Phase 4 - resting membrane potential
R-HSA-1483206: Glycerophospholipid biosynthesis
R-HSA-9008059: Interleukin-37 signaling
R-HSA-5675221: Negative regulation of MAPK pathway
R-HSA-210500: Glutamate Neurotransmitter Release Cycle
R-HSA-6791312: TP53 Regulates Transcription of Cell Cycle Genes
0 2 4 6 8 10
-log10(P)

Supplementary Fig. 14 | Pathway analysis. a, Top 20 differentially regulated Reactome pathways between the diabetic condition and untreated control on D14. As an input, differentially regulated genes with an absolute Log2 FC > 1 were selected. **b**, Top 20 Reactome pathways on D28.

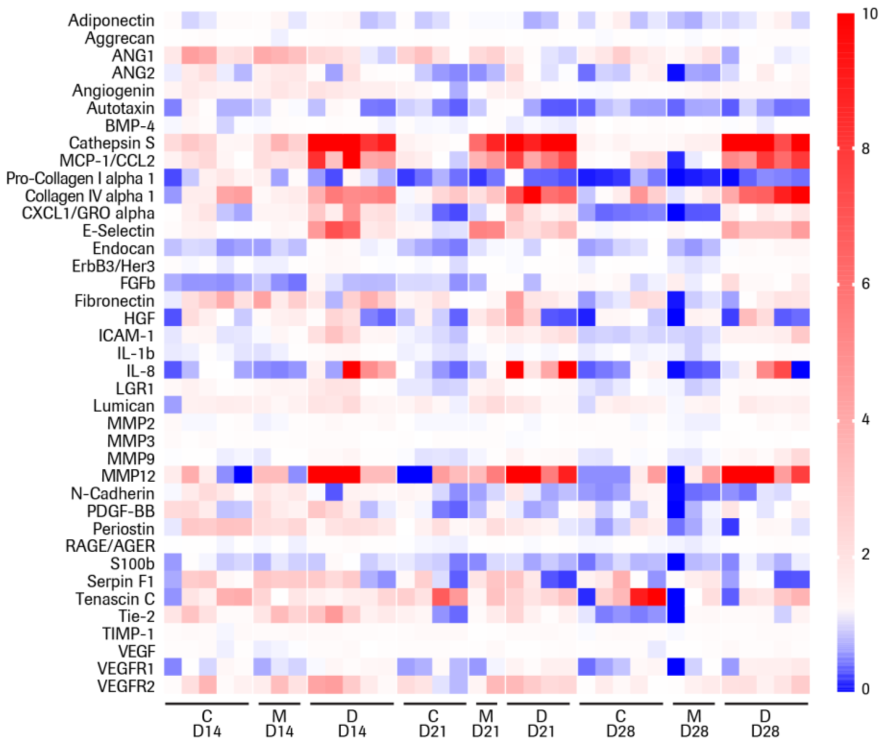

Supplementary Fig. 15 | Diabetic analyte measurements. Heat map of analyte measurements on untreated control, osmotic control and diabetic supernatants on D14, D21 and D28, showing analyte concentrations normalized to values on D7. The scale indicates fold change to control. Supernatants were obtained from n = 5 independent experiments. Source data are provided as a Source Data file.

236

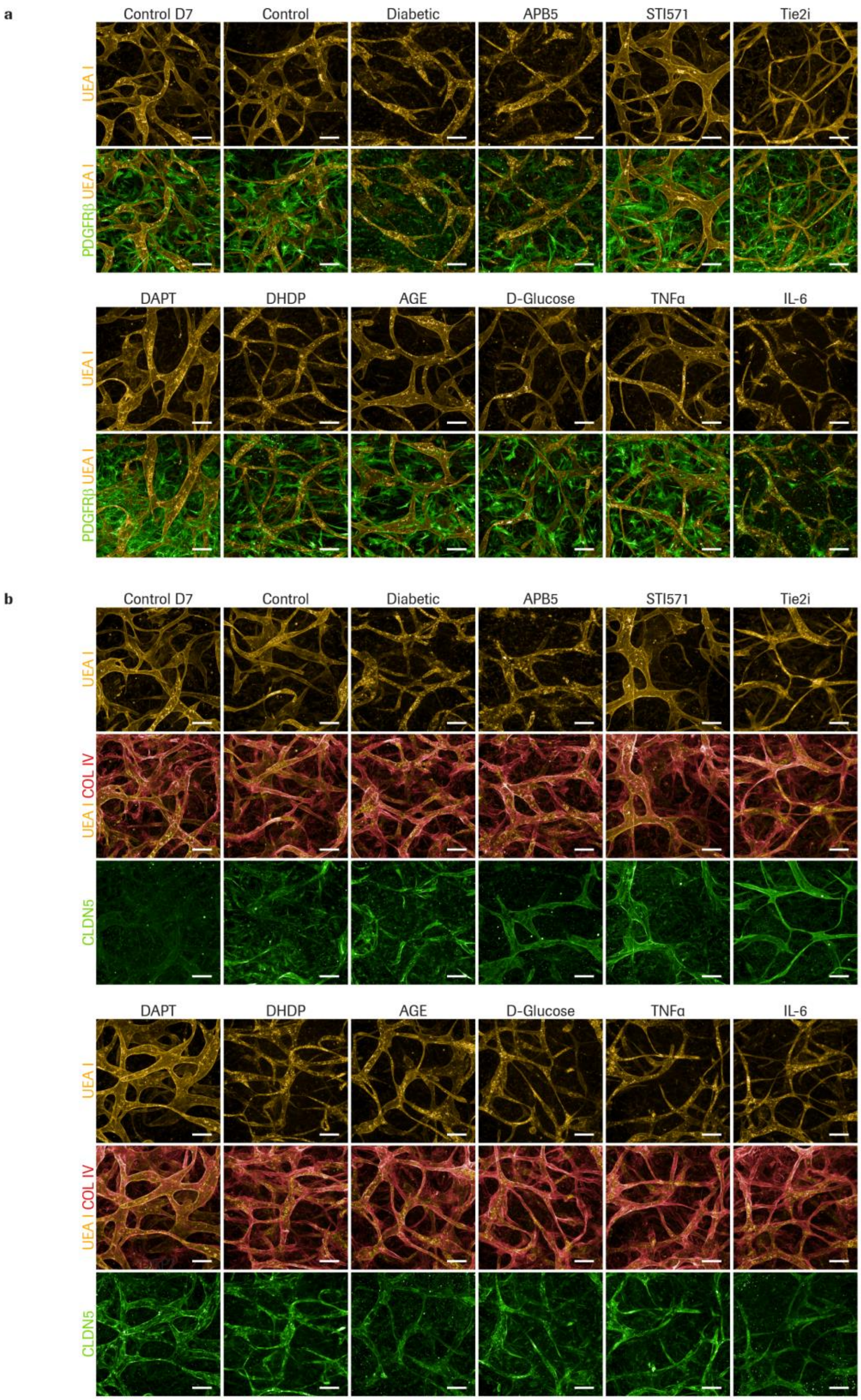
a
Control D7
Control
Diabetic
APB5
STI571
Tie2i
UEA I
PDGFRβ UEA I
DAPT
DHDP
AGE
D-Glucose
TNFα
IL-6
b
UEA I COL IV
CLDN5

237

Supplementary Fig. 16 | Inhibition of pericyte-endothelial cell communication pathways cause microvascular alterations. a, Representative images of endothelial networks (UEA I) and overlay images with pericytes (PDGFRβ) on D14, following 7 days of treatment. Controls are shown after iBRB MVN formation on D7 (Control D7) and on the treatment endpoint D14 (Control). All treatments were applied from D7 to D14. STI571, PDGFRβ inhibitor. Treatments with D-Glucose, TNF-α or IL-6 were tested individually, and in combination to produce the diabetic treatment (Diabetic). **b**, Representative images of iBRB MVNs (UEA I), overlay images with basement membranes (COL IV), and images of tight junctions (CLDN5) on D14, following 7 days of treatment. Images show maximum intensity projections of 395 μm Z-stacks, from n = 2 independent experiments and n = 1 experiment for D-Glucose, TNF-α and IL-6 conditions. Scale bars, 100 μm (**a**, **b**).

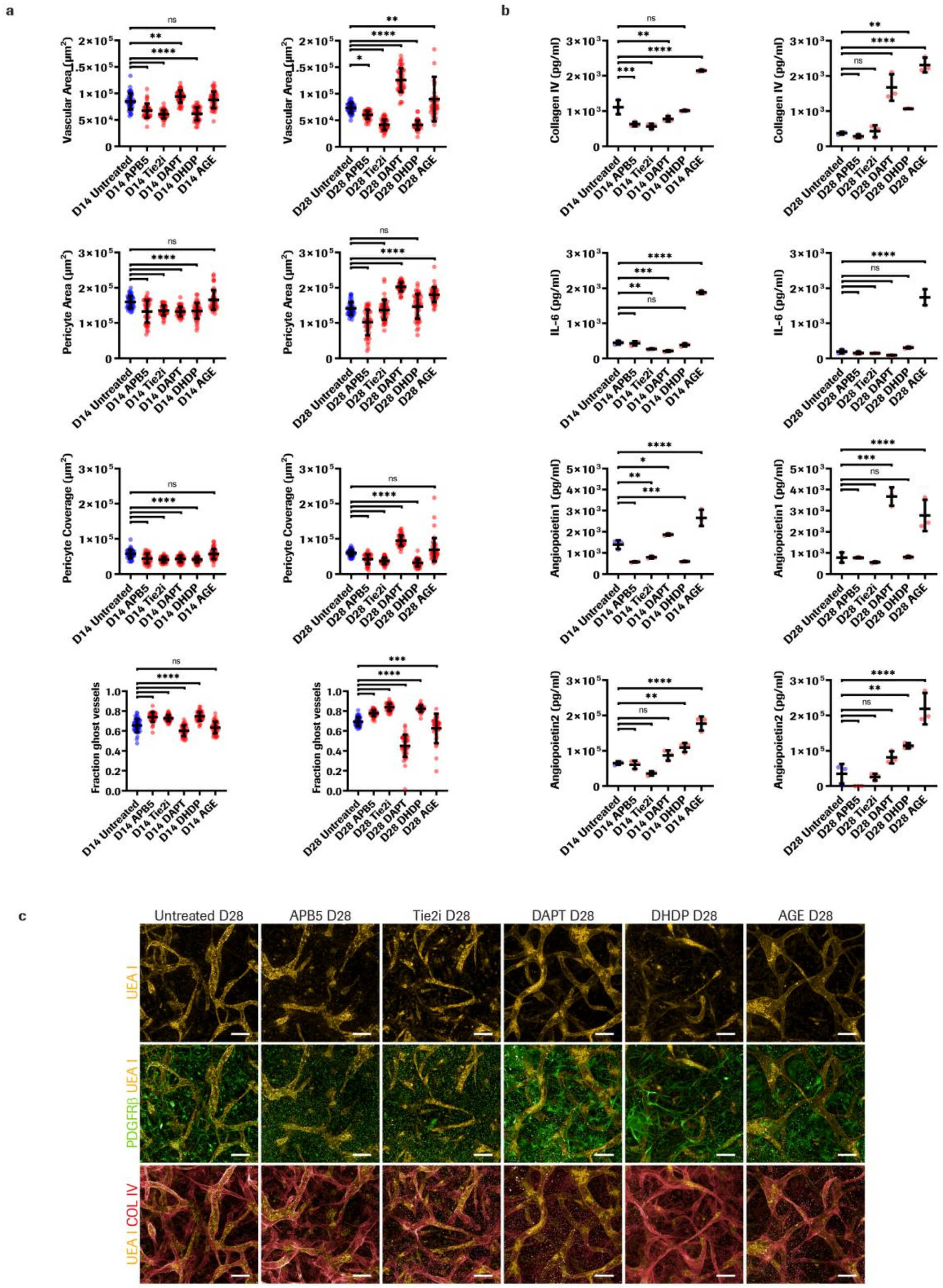

Supplementary Fig. 17 | Long-term treatment effects. a, Quantification of vascular area, pericyte area and coverage, and fraction ghost vessels for untreated, APB5, Tie2i, DAPT, DHDP

and AGE-treated conditions on D14 and D28. n = 45 untreated D14, n = 44 D28, n = 43 APB5 D14, n = 41 D28, n = 44 Tie2i D14, n = 45 D28, n = 44 DAPT D14, n = 45 D28, n = 44 DHDP D14, n = 45 D28, n = 45 AGE D14 and n = 45 D28 treated ROI networks analyzed from n = 3 replicate channels. **b**, Analyte measurements of human collagen IV alpha I, IL-6, Angiopoietin-1 and Angiopoietin-2 obtained from n = 3 replicate channels. Data are mean ± s.d. *P<0.05; **P<0.01; ***P<0.001; ****P<0.0001; one-way ANOVA. Source data are provided as a Source Data file. **c**, Representative images of endothelial networks (UEA I) and overlay images with pericytes (PDGFRβ) or basement membranes (COL IV) on D28. All images show maximum intensity projections of 245 μm Z-stacks. Scale bars, 100 μm (**c**).

a

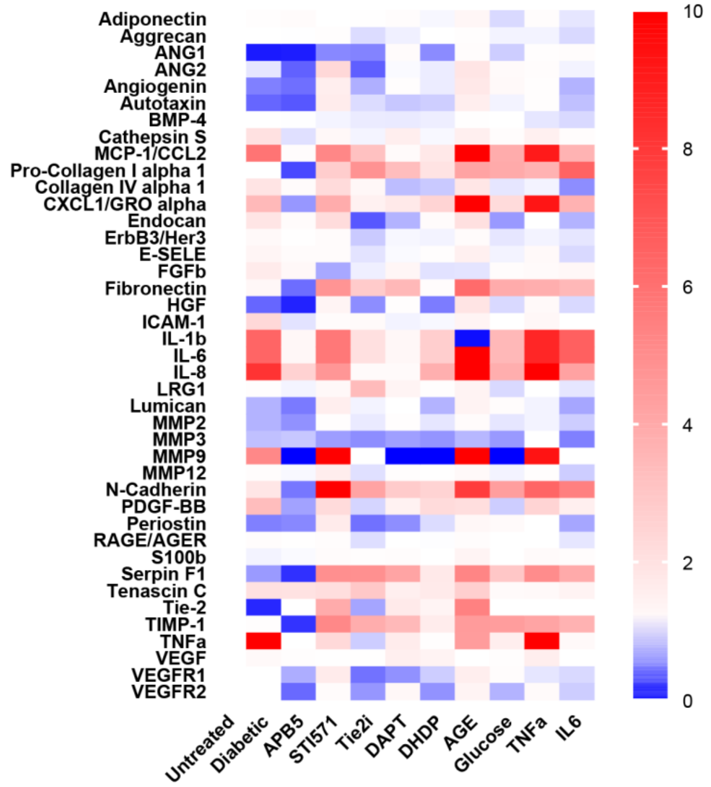
Adiponectin
Aggrecan
ANG1
ANG2
Angiogenin
Autotaxin
BMP-4
Cathepsin S
MCP-1/CCL2
Pro-Collagen I alpha 1
Collagen IV alpha 1
CXCL1/GRO alpha
Endocan
ErbB3/Her3
E-SELE
FGFb
Fibronectin
HGF
ICAM-1
IL-1b
IL-6
IL-8
LRG1
Lumican
MMP2
MMP3
MMP9
MMP12
N-Cadherin
PDGF-BB
Periostin
RAGE/AGER
S100b
Serpin F1
Tenascin C
Tie-2
TIMP-1
TNFa
VEGF
VEGFR1
VEGFR2
Untreated
Diabetic
APB5
STI571
Tie2i
DAPT
DHDP
AGE
Glucose
TNFa
IL6
10
8
6
4
2
0

b

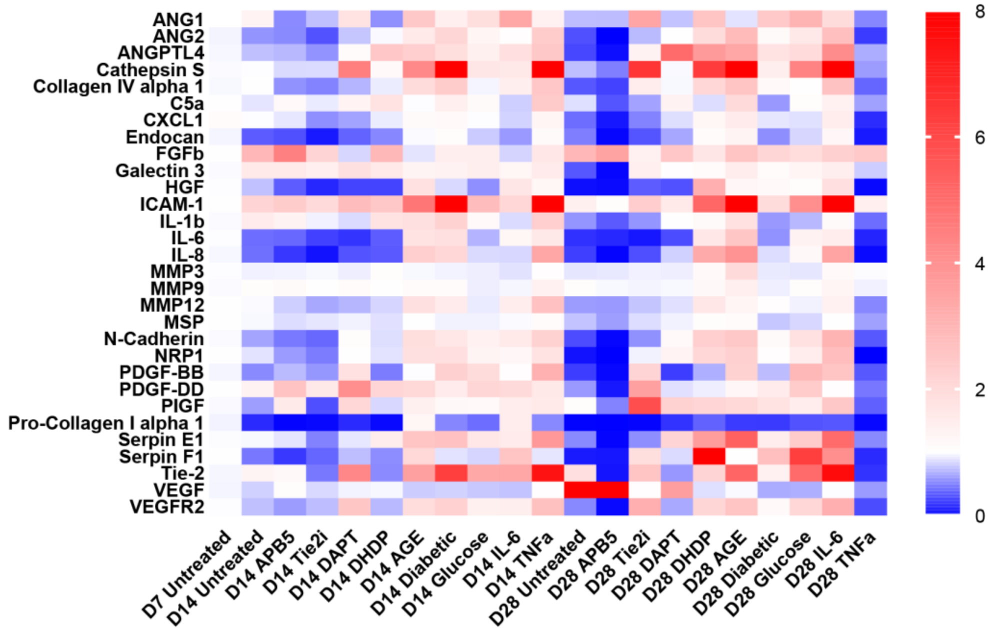
ANG1
ANG2
ANGPTL4
Cathepsin S
Collagen IV alpha 1
C5a
CXCL1
Endocan
FGFb
Galectin 3
HGF
ICAM-1
IL-1b
IL-6
IL-8
MMP3
MMP9
MMP12
MSP
N-Cadherin
NRP1
PDGF-BB
PDGF-DD
PlGF
Pro-Collagen I alpha 1
Serpin E1
Serpin F1
Tie-2
VEGF
VEGFR2
D7 Untreated
D14 Untreated
D14 APB5
D14 Tie2i
D14 DAPT
D14 DHDP
D14 AGE
D14 Diabetic
D14 Glucose
D14 IL-6
D14 TNFa
D28 Untreated
D28 APB5
D28 Tie2i
D28 DAPT
D28 DHDP
D28 AGE
D28 Diabetic
D28 Glucose
D28 IL-6
D28 TNFa
8
6
4
2
0

Supplementary Fig. 18 | Treatments analyte measurements. a, Heat map of analyte measurements for untreated, diabetic, APB5, STI571 (PDGFRβ inhibitor), Tie2i, DAPT, DHDP, AGE, glucose, TNF-α and IL-6-treated conditions on D14, normalized to the untreated control on D14. STI571, PDGFRβ inhibitor. Supernatants were pooled from n = 3 replicate channels. **b**, Heat map of analyte measurements for untreated conditions on D7, D14 and D28, and APB5, Tie2i, DAPT, DHDP, AGE, diabetic, glucose, IL-6 and TNF-α-treated conditions on D14 and D28, normalized to the untreated control on D7. The scale indicates fold change to control. Supernatants were obtained from n = 3 replicate channels. Source data are provided as a Source Data file.

Supplementary Table 1 | Information about cells used in this study.

Cell type	Source	Type	Supplier	Cat number	Batch number	Donor information
HRMVEC - 1	retina	primary	Cell Sytems	ACBRI 181	181.04.01.02.02	NA
HRMVEC - 2	retina	primary	Pelo-Biotech	PB-CH-160-8511	QC-18B19F09	28 year-old female
HRMVEC - 3	retina	primary	Pelo-Biotech	PB-CH-160-8511	QC-03B18F04	NA
HRMVEC - 4	retina	primary	Cell Biologics	H-6065	F021518Ag	NA
HRMVEC - 5	retina	primary	Cell Biologics	H-6065	M021518	NA
HRMVEC - 6	retina	primary	Cell Biologics	H-6065	122118U	NA
HRMVEC - 7	retina	primary	Cell Biologics	H-6065	120117Ag	NA
HRP	retina	primary	Cell Sytems	ACBRI 183	183.02.01.01.10	NA
HRA - 1	retina	primary	ScienCell	1870	23521	20 week-old male
HRA - 2	retina	primary	ScienCell	1870	30020	56 year-old female
HCA	brain	immortalized	Innoprot	P10251-IM	NA	NA

NA: not provided by the supplier
