# Supplementary material for: Modeling early pathophysiological phenotypes of diabetic retinopathy in a human inner blood-retinal barrier-on-a-chip
Source: Nat Commun. 2024 Feb 14;15:1372. doi: 10.1038/s41467-024-45456-z (PMC10866954; doi:10.1038/s41467-024-45456-z)
Supplement: Supplementary file 3 — Reporting Summary [file 41467_2024_45456_MOESM3_ESM.pdf]

nature portfolio

Corresponding author(s): Héloïse Ragelle, Roger D. Kamm

Last updated by author(s): Mar 29, 2023

Reporting Summary

Nature Portfolio wishes to improve the reproducibility of the work that we publish. This form provides structure for consistency and transparency in reporting. For further information on Nature Portfolio policies, see our Editorial Policies and the Editorial Policy Checklist.

Statistics

For all statistical analyses, confirm that the following items are present in the figure legend, table legend, main text, or Methods section.

n/a	Confirmed	
☐	☒	The exact sample size (*n*) for each experimental group/condition, given as a discrete number and unit of measurement
☐	☒	A statement on whether measurements were taken from distinct samples or whether the same sample was measured repeatedly
☐	☒	The statistical test(s) used AND whether they are one- or two-sided<br>*Only common tests should be described solely by name; describe more complex techniques in the Methods section.*
☒	☐	A description of all covariates tested
☒	☐	A description of any assumptions or corrections, such as tests of normality and adjustment for multiple comparisons
☐	☒	A full description of the statistical parameters including central tendency (e.g. means) or other basic estimates (e.g. regression coefficient) AND variation (e.g. standard deviation) or associated estimates of uncertainty (e.g. confidence intervals)
☐	☒	For null hypothesis testing, the test statistic (e.g. *F*, *t*, *r*) with confidence intervals, effect sizes, degrees of freedom and *P* value noted<br>*Give P values as exact values whenever suitable.*
☒	☐	For Bayesian analysis, information on the choice of priors and Markov chain Monte Carlo settings
☒	☐	For hierarchical and complex designs, identification of the appropriate level for tests and full reporting of outcomes
☒	☐	Estimates of effect sizes (e.g. Cohen's *d*, Pearson's *r*), indicating how they were calculated

Our web collection on statistics for biologists contains articles on many of the points above.

Software and code

Policy information about availability of computer code

Data collection	Harmony software (v5.1), xPonent software (v4.2)
Data analysis	KNIME Analytics Platform (v4.5.1), Microsoft Excel 2016 (v16.0), GraphPad Prism 8 (v8.4.2), Metascape (v3.5, https://metascape.org)

For manuscripts utilizing custom algorithms or software that are central to the research but not yet described in published literature, software must be made available to editors and reviewers. We strongly encourage code deposition in a community repository (e.g. GitHub). See the Nature Portfolio guidelines for submitting code & software for further information.

Data

Policy information about availability of data

All manuscripts must include a data availability statement. This statement should provide the following information, where applicable:

- Accession codes, unique identifiers, or web links for publicly available datasets
- A description of any restrictions on data availability
- For clinical datasets or third party data, please ensure that the statement adheres to our policy

Source data are provided with this paper. All raw data needed for quantification are available in the source data files. Raw image files, differential gene expression analysis files and other resources are available on request from the corresponding author. The transcriptomic data set is available through GEO under accession number GSE228900.

Human research participants

Policy information about studies involving human research participants and Sex and Gender in Research.

Reporting on sex and gender	n/a
Population characteristics	n/a
Recruitment	n/a
Ethics oversight	n/a

Note that full information on the approval of the study protocol must also be provided in the manuscript.

Field-specific reporting

Please select the one below that is the best fit for your research. If you are not sure, read the appropriate sections before making your selection.

☒ Life sciences ☐ Behavioural & social sciences ☐ Ecological, evolutionary & environmental sciences

For a reference copy of the document with all sections, see nature.com/documents/nr-reporting-summary-flat.pdf

Life sciences study design

All studies must disclose on these points even when the disclosure is negative.

Sample size	Primary cells from different donors were used. Morphology of endothelial networks generated from multiple donors was compared to assess donor variability. No sample size calculation was performed.
Data exclusions	No data were excluded from the analyses.
Replication	Experiments were repeated 3 times independently, unless stated otherwise, from new frozen cell vials for each cell type. Images were acquired of whole channels per device, and multiple devices per experimental condition. All attempts at replication were successful.
Randomization	Randomization was not used since there were no participants and study results could not be influenced by bias.
Blinding	Blinding was not used since there were no participants and study results could not be influenced by bias.

Reporting for specific materials, systems and methods

We require information from authors about some types of materials, experimental systems and methods used in many studies. Here, indicate whether each material, system or method listed is relevant to your study. If you are not sure if a list item applies to your research, read the appropriate section before selecting a response.

Materials & experimental systems

n/a	Involved in the study	
☐	☒	Antibodies
☐	☒	Eukaryotic cell lines
☒	☐	Palaeontology and archaeology
☒	☐	Animals and other organisms
☒	☐	Clinical data
☒	☐	Dual use research of concern

Methods

n/a	Involved in the study	
☒	☐	ChIP-seq
☒	☐	Flow cytometry
☒	☐	MRI-based neuroimaging

Antibodies

Antibodies used	Anti-CD31 (Abcam, ab24590), anti-PDGFRβ (Abcam, ab32570), anti-S100b (Sigma, S2532), anti-desmin (ab15200), anti-GFAP (Invitrogen, MA5-12023), anti-CLDN5 (Invitrogen, 341600), anti-ZO-1 (Thermo Fisher, 339100), anti-VE-cadherin (Abcam, ab33168), anti-laminin (Abcam, ab7463), anti-collagen type IV (Sigma, MAB1910), Alexa Fluor 488 donkey anti-rabbit (Invitrogen, A-21206), Alexa Fluor 647 donkey anti-mouse (Invitrogen, A-31571).
Validation	Validation of the primary antibodies was based on the data included in the manuscript.

Eukaryotic cell lines

Policy information about cell lines and Sex and Gender in Research

Cell line source(s)	All cells used are primary human retinal microvascular cells obtained from commercial sources.<br>Human Retinal Microvascular Endothelial Cells HRMVEC (Cell Systems ACBRI 181; Pelo-Biotech PB-CH-160-8511; Cell Biologics H-6065)<br>Human Retinal Pericytes HRP (Cell Systems ACBRI 183)<br>Human Retinal Astrocytes HRA (ScienCell 1870)<br>Immortalized Human Cortical Astrocytes HCA (Innoprot P10251-IM)
Authentication	Cells were authenticated with immunofluorescence of cell type specific markers.
Mycoplasma contamination	Cells tested negative for mycoplasma contamination.
Commonly misidentified lines<br>(See ICLAC register)	No commonly misidentified cell lines were used in the study.
